# Genomic Characterization and Phylogenetic Analysis of Five Avian Influenza H5N1 Subtypes from Wild *Anser indicus* in Yunnan, China

**DOI:** 10.3390/vetsci12030280

**Published:** 2025-03-17

**Authors:** Lingsi Yang, Rui Wang, Qi Liu, Taif Shah, Jiuxuan Zhou, Wenhua Zhao, Yixuan Wang, Lulu Deng, Binghui Wang

**Affiliations:** 1 Faculty of Life Science and Technology, Kunming University of Science and Technology, Kunming 650500, China; lingsiyang@aliyun.com (L.Y.); larrywangrui@aliyun.com (R.W.); taifshah@yahoo.com (T.S.); 3055779157@aliyun.com (L.D.); 2 Yunnan Province Key Laboratory of Public Health and Biosafety, School of Public Health, Kunming Medical University, Kunming 650500, China; liuqi2401@163.com; 3 Research Institute of Forest Protection, Yunnan Academy of Forestry and Grassland, Kunming 650500, China; jiuxuan_zhou@163.com (J.Z.); yixuan_wang201804@163.com (Y.W.); 4 Yunnan Tropical and Subtropical Animal Viral Disease Laboratary, Yunnan Animal Science and Veterinary Institute, Kunming 650224, China; tigerhua74@aliyun.com

**Keywords:** *Anser indicus*, highly pathogenic avian influenza virus, clade 2.3.4.4b, characterization, homology

## Abstract

Highly pathogenic avian influenza (HPAI) H5 viruses have been found to have a substantial geographic distribution since they were first reported in Guangdong Province, China. The emergence of new genotypes threatens the poultry industry and human health worldwide. Here, we report five HPAI H5N1 variants isolated from *Anser indicus* in Yunnan Province, China. A phylogenetic analysis of the hemagglutinin (HA) gene showed that all isolates belong to the highly pathogenic H5 clade 2.3.4.4b and formed two distinct genetic clusters. Bayesian phylogenetic analysis also revealed that the viruses were initially disseminated from wild birds to *Anser indicus*, implying that infected birds most likely contributed to viral transmission in the region. Genomic sequence analysis revealed several amino acid substitutions, also implying that the infected birds contributed to the spread of the virus throughout the region. Substitutions in the HA glycoprotein increased the virus’s binding affinity to human α-2,6 sialic acid residues. Substitutions in the PB1, PA, and PB2 motifs increased viral polymerase activity and replication in hosts, whereas substitutions in the NP, M1, and NS motifs increased viral pathogenicity in chickens and mice.

## Simple Summary

The geographical conditions of Yunnan Province, including its diverse climate and ecosystems and its role as an important stopover for migratory birds in Asia, make the province a key player in the transmission of avian influenza. The migratory activities of wild birds are not only a pathway for virus transmission but also closely linked to the province’s efforts to prevent and control avian influenza. The genetic diversity of the H5N1 that we have reported highlight the importance of systemic surveillance and risk assessment in wild migratory birds and domestic poultry before the virus evolves in humans.

## 1. Introduction

Influenza A viruses (IAVs) are single-stranded RNA viruses with eight gene segments that are divided into subtypes based on the antigenicity of their surface glycoproteins: 18 hemagglutinin (HA) and 11 neuraminidase (NA) [1]. Avian influenza viruses (AIVs) are further classified as low pathogenic avian influenza (LPAI) or highly pathogenic avian influenza (HPAI) based on their virulence. LPAI viruses typically present few or no clinical signs. In contrast, HPAI viruses (specifically H5 and H7 subtypes A) are highly contagious, causing systemic diseases in wild birds, poultry, and humans with high mortality [2]. Wild aquatic birds, mainly migratory waterfowl, have a large IAV genetic pool and are considered natural reservoirs of these viruses [3].

Since their first appearance in Guangdong, China, in 1996 [4], highly pathogenic avian influenza (HPAI) viruses of the H5N1 subtype have spread widely across Asia, Europe, and Africa, infecting a range of domestic and wild birds [5], other mammals [6,7], and humans [8]. Over time, the HPAI H5 lineage has evolved into multiple phylogenetically distinct sub-lineages, with branch 2.3.4.4 gaining attention for the first time in 2014 and subsequently emerging as globally dominant [9]. These viruses have extensive recombination and transmission capabilities and can produce H5Nx viruses, especially H5N2, H5N5, H5N6, and H5N8 [10], with various gene clusters. It is possible that poultry belonging to the family Chickenidae that were infected with LPAI viruses may have caused these to evolve into HPAI viruses through amino acid substitutions at cleavage sites or non-homologous recombination with other viral genes [11]. These viruses have caused large-scale outbreaks on several continents, resulting in the culling of an estimated 49 million poultry animals, causing significant economic losses to the poultry industry and a global public health and safety issue that poses a major threat to the health of humans and other domestic mammals.

Yunnan Province is located on the southwestern border of China and has abundant genetic resources and a large number of poultry breeds. However, due to relatively inconvenient transportation and a lack of development, the state of avian influenza prevention and control in Yunnan Province is quite severe. In addition, Yunnan Province borders multiple countries and there is a risk of cross-border transmission of the avian influenza virus [12,13,14]. Yunnan has several wintering sites in addition to the Dashanbao Nature Reserve, where millions of migrating birds come into direct or indirect contact with domestic waterfowl annually, which may promote AIV transmission [15]. Therefore, conducting investigations on avian influenza viruses is of great significance for the timely understanding of epidemic dynamics, assessing infection risks, and formulating prevention and control strategies. In 2021, The National Forestry and Grassland Administration identified five dead *Anser indicus* in the Dashanbao Dahaizi Nature Reserve, Yunnan, China. Lung and tracheal tissue samples were collected from each dead bird. Five H5N1 isolates were identified from the dead *Anser indicus* tissue samples using polymerase chain reaction (PCR). In this study, we performed gene characterization and a phylogenetic analysis based on the sequences of the eight genomic segments to better understand the evolution and genetic diversity of the newly identified H5N1 variants.

## 2. Materials and Methods

On 1 December 2021, five dead *Anser indicus* individuals were collected from the Dashanbao Nature Reserve, Yunnan Province, China. All fresh dead birds were transported to the biosafety laboratory on dry ice. Visceral tissue samples were collected, immersed in a viral transport medium, and stored at −80 °C until further processing.

For RNA extraction, lung and other tissue samples were mixed and homogenized with Qiangen TissueLyser. Then, the supernatants containing viral RNA were subjected to RNA extraction using the TIANamp virus RNA kit (DP315-R, Beijing, China). AIV genomes were amplified from the extracted RNA using universal gene-specific primers [16] and an Ex Taq polymerase (TAKARA, Kyoto, Japan), and the amplified PCR product was selected on a 1% agarose gel for library construction. Sequencing libraries were generated using the ALFA-SEQ DNA Library Prep Kit for Illumina (FINDROP, Guangzhou, China) following the manufacturer’s guidelines. The library quality was assessed using the Qubit dsDNA HS Assay Kit (Thermo Fisher Scientific, Waltham, MA, USA) before sequencing on an Illumina Novaseq 6000 system (Illumina, San Diego, CA, USA) [17].

The genomic sequences retrieved from the NCBI Influenza Virus Database were used to construct the phylogenetic tree. The nucleotide sequences of each fragment were aligned with the MAFFT tool, and duplicate and missing sequences were removed. The maximum likelihood phylogenetic trees were generated for the eight genomic fragments using MEGA-X 10.2 software, which implemented the best-fit substitution model over 10,000 ultrafast bootstraps [18]. The best-fit substitution model was selected using the Bayesian information criterion of ModelFinder and implemented in MEGA-X 10.2 software. Meanwhile, the obtained whole-genome sequences were translated into protein sequences using MEGA-X and compared with previously reported key amino acid sites to determine whether mutations existed at these positions.

## 3. Results and Discussion

In this study, we identified five HPAI H5 viruses from dead *Anser indicus* with obvious gross lesions of concern in the heart, trachea, stomach, and other organs. Details of the lesions are shown in Supplementary Table S1.

Our genomic homology analysis showed that the similarity of the whole-genome sequences of five H5N1 ranged from 93.3% to 100%, which suggests a relatively close evolutionary link between them, implying that these virus strains have retained stable genetic traits throughout their transmission, likely originating from a common ancestral virus. Details of the nucleotide sequence homology are shown in Supplementary Table S2.

**Figure 1:**
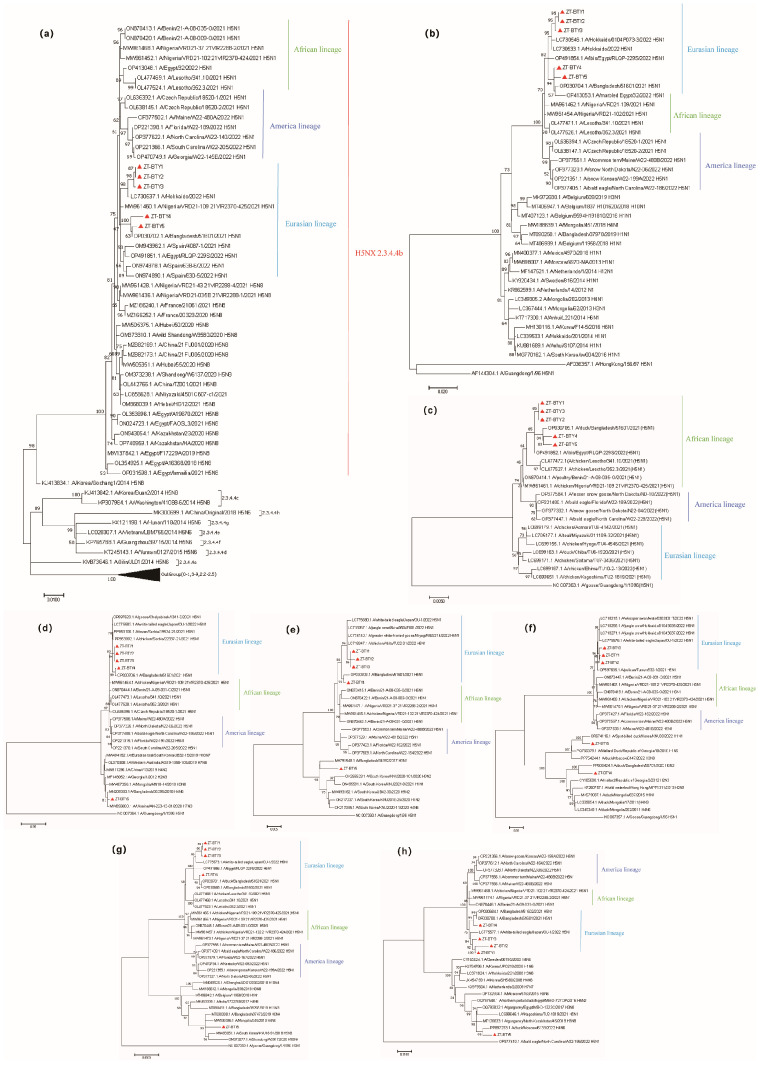
Phylogenetic analysis of the five highly pathogenic avian influenza H5N1 viruses from migratory dead Anser indicus in Yunnan, China. Annotation: The coding region of H5N1 hemagglutinin (HA) was used for phylogenetic tree reconstruction (**a**): the phylogenetic trees of NA (**b**) and M (**c**), NS (**d**), NP (**e**), PB2 (**f**), PA (**g**), and PB1 (**h**) were generated using the maximum likelihood method with MEGA-X 10.2 software. The H5N1 gene sequence obtained in this study is marked with a red triangle, while the remaining black parts are reference sequences downloaded from the NCBI database. Different colors on the right-hand side indicate different lineages. The scale bar represents the distance between sequence pairs and the horizontal distances are proportional to the genetic distance.

Nucleotide sequence analysis of the complete *HA* gene showed that the five H5N1 viruses belonged to clade 2.3.4.4b and clustered with H5N1 viruses previously isolated from Africa and Asia. Most H5 avian influenza viruses (AIVs) identified during 2020–2022 were in that clade, while H5N8 was the dominant subtype during 2019–2021, and H5N1 strains emerged in October 2020 and increased from that point on. Three isolates formed a cluster with H5N1 variants from Hokkaido, Japan. In contrast, the other two isolates clustered with the H5N1 virus from Bangladesh (Figure 1a), suggesting that the same virus was introduced in different countries. The phylogenetic analysis showed that except for the M gene belonging to the African lineage (Figure 1c), all other genes belong to the Eurasian lineage (Figure 1b,d–h), suggesting the possibility of genome recombination in the avian influenza virus.

Except for the A/Anser indicus/China/ZT-BTY5/2021(H5N1), all fragments of the other four avian influenza strains obtained in this study clustered with H5Nx viruses prevalent on the Eurasian continent from 2021 to 2022. The phylogenetic analysis of the NS, NP, PB1, PB2, and PA genes showed that the five strains of H5N1 are divided into two distinct groups; four of these strains clustered together and belong to the highly pathogenic H5N1 internal genes, whereas the remaining single strain clustered with the low pathogenic avian influenza viruses. This indicates that the genome of the A/Anser indicus/China/ZT-BTY5/2021(H5N1) represents a reassortment of the 2021–2022 Eurasian H5Nx (HA, NA) with LPAIVs (NS, NP, PA, PB1, PB2,) prevalent in Asia and Europe. This suggests a strong link between the viruses identified in Yunnan and foreign strains, likely generated through the recombination of various strains from different countries. In a previous study, clade 2.3.4.4b viruses were divided into clusters A and B [19]. In this study, all H5N1 variants originated from cluster A, implying that cluster A is stable in Yunnan. Given that HPAI H5N1 is likely to continue to spread among wild birds, poultry vaccination is highly recommended to prevent viral outbreaks. In addition, overwintering and stopover sites along migration routes should be continuously monitored for AIVs. In 2020, six H5 variants (one H5N2, two H5N3, and three H5N8) were identified during the regular surveillance of AIVs from wild birds in China [20]. These viruses are classified as LPAIs, but, when different strains co-infect the same host, and the resulting strains, which have different gene constellations, may have different biological characteristics [21,22]. It is recommended that the number of infected populations in the region be reduced to minimize the likelihood that they will evolve into HPAIs.

The HA glycoproteins of the five H5N1 viruses all contained REKRRKR↓GLF motifs at the cleavage site, indicating that they were highly pathogenic. All receptor-binding sites of the isolates at positions 222–224 (H5 numbering) were QRG; however, amino acid substitutions in HA glycoproteins can enhance the binding affinity between viruses and human alpha-2,6 sialic acid residues, which might increase their transmission to other mammals [23]. Meanwhile, the adaptation of viral polymerase to the host is crucial for the inter-species transmission of H5N1. Existing evidence suggests that mutations in PB2 increase the virus’s replication ability and adaptability in mammals, leading to high pathogenicity [24]. Mutations in PB1 and PA protein sites promote their polymerase activity in duck and mammalian cell lines, enhancing virus replication [25,26,27]. Additionally, the M gene plays multiple roles in the invasion of avian influenza viruses into hosts. Mutations in M1 and NP proteins have been proven to play an important role in enhancing the adaptability of viruses [28,29,30], while M2 and NA proteins are considered to be associated with host markers and virus resistance [31,32,33,34]. NS1 plays a crucial role in disrupting the antiviral immune response of host cells [29]. Specific amino acid site information is shown in Table 1.

In our study, the amino acid substitutions (T160A, S128P, S137A) in the HA motif increased the risk of virus transmission to other mammals [21]: L89V (in the PB2 segment); L473V (in the PB1 segment); and N383D, N409S, and S515T (in the PA segment) were attributed to the increase in viral polymerase activity in mammals (Table 1), which is consistent with previous findings [27], implying that understanding amino acid substitutions can provide insights into potential zoonotic mechanisms. Amino acid substitutions (N30D, I43M, and T215A) in the M1 motif may contribute to increasing viral pathogenesis in mice, which is consistent with the previous finding that substitutions in the M1 protein contribute to H5N1 virulence [30]. Furthermore, P42S substitutions in the NS protein can increase viral virulence in mammals [27,39]. These findings suggest the need for the systematic surveillance of LPAI H5 viruses in overwintering sites to provide an early warning of viral evolution into HPAI variants.

In conclusion, as an important transit station for Asian migratory birds, Yunnan Province is a key participant in the spread of avian influenza. Our report of H5N1-associated genetic diversity highlights the importance of systemic surveillance and risk assessments of wild migratory birds and domestic poultry before the virus evolves in humans.

## Figures and Tables

**Table 1:** Amino acid sequences and substitutions in HPAI H5N1 viruses isolated from wild dead *Anser indicus* in Yunnan, China.

Protein	Amino Acid/Motif	Phenotypic Characteristic	ZT-BTY-1	ZT-BTY-2	ZT-BTY-3	ZT-BTY-4	ZT-BTY-5
**HA**	N158D	Lack of glycosylation sites to promote airborne transmission to ferrets and humans [35]	N	N	N	D	D
	T160A		A	A	A	A	A
	S128P	Enhanced sialic acid A-2, 6-galactose receptor tropism [36]	P	P	P	P	P
	S137A		A	A	A	A	A
	Q226L		Q	Q	Q	Q	Q
	S227R		R	R	R	R	R
	G228S		G	G	G	G	G
**NA**	E119V	Neuraminidase inhibitor resistance [31,32,33]	E	E	E	E	E
	R152K		R	R	R	R	R
	H275Y		H	H	H	H	H
	R293K		R	R	R	R	R
	N295S		N	N	N	N	N
	I117T		I	I	I	I	I
**PB2**	L89V	Increased virus polymerase activity [25]	V	V	V	V	V
	E627K	Mammalian tropism [37,38]	E	E	E	E	E
	M631L		M	M	M	M	M
	D701N		D	D	D	D	D
**PB1**	L473V	Increased virus polymerase activity and replication in mammalian cell lines [27]	V	V	V	V	V
	L598P		L	L	L	L	L
**PA**	N383D	Increased virus polymerase activity and replication in duck and mammalian cell lines [27]	D	D	D	D	D
	N409S		S	S	S	S	S
	K497R		K	K	K	K	K
	S515T		T	T	T	T	T
**NP**	M105V	Increased virus pathogenicity in chickens [29]	V	V	V	V	V
	I109T		I	I	I	I	I
**M1**	N30D	Increased virus pathogenicity in mice [29,30]	D	D	D	D	D
	I43M		M	M	M	M	M
	T215A		A	A	A	A	A
**M2**	L26F	M2 ion channel inhibitor resistance [34]	L	L	L	L	L
	S31N		S	S	S	S	S
**NS**	P42S	Enhanced virus pathogenicity in mice [25,29]	S	S	S	S	S

HA: hemagglutinin; NA: Neuraminidase; PB2: Polymerase basic 2; PB1: Polymerase basic 1; PA: Polymerase acidic 1; NP: Nucleoprotein; M1/M2: Matrix protein; NS: Nonstructural protein.

## Data Availability

The full-length genome segments of the five H5N1 viruses have been deposited in the NCBI database under the following accession numbers: **A/Anser indicus/China/ZT-BTY1/2021(H5N1): ZT-BTY1** (PB2: PP032037, PB1: PP032038, PA: PP032039, HA: PP031948, NP: PP032040, NA: PP031948, M: PP052966, NS: PP032042). **A/Anser indicus/China/ZT-BTY2/2021(H5N1): ZT-BTY2** (PB2: PP032050, PB1: PP032051, PA: PP032052, HA: PP031949, NP: PP032053, NA: PP032054, M: PP053018, NS: PP032055). **A/Anser indicus/China/ZT-BTY3/2021(H5N1): ZT-BTY3** (PB2: PP032836, PB1: PP032837, PA: PP032838, HA: PP031950, NP: PP032839, NA: PP032840, M: PP053019, NS: PP032841). **A/Anser indicus/China/ZT-BTY4/2021(H5N1): ZT-BTY4** (PB2: PP032855, PB1: PP032856, PA: PP032857, HA: PP031951, NP: PP032858, NA: PP032859, M: PP053020, NS: PP032860). **A/Anser indicus/China/ZT-BTY5/2021(H5N1): ZT-BTY5** (PB2: PP032861, PB1: PP032862, PA: PP032863, HA: PP031952, NP: PP032864, NA: PP032865, M: PP053021, NS: PP032866).

## Supplementary Materials

The following supporting information can be downloaded at https://www.mdpi.com/article/10.3390/vetsci12030280/s1, Table S1. Detection of HPAI H5N1 viruses and their symptoms in dead migratory Anser indicus in Yunnan, China; Table S2. Homology of eight HPAI H5N1 genes from Anser indicus and the most similar sequences in NCBI GenBank.
