# Supplementary material for: Genomic Characterization and Phylogenetic Analysis of Five Avian Influenza H5N1 Subtypes from Wild Anser indicus in Yunnan, China
Source: Vet Sci. 2025 Mar 17;12(3):280. doi: 10.3390/vetsci12030280 (PMC11945867; doi:10.3390/vetsci12030280)
Supplement: Supplementary file 1 [file vetsci-12-00280-s001.zip › vetsci-3449999-supplementary.pdf]

Supplementary information.

Table S1. Detection of HPAI H5N1 viruses and their symptoms in dead migratory *Anser indicus* in Yunnan China.

Isolates	Viral strain ID	Amino acid Substitutions at the HA cleavage site	Observed symptoms and gross lesions
A/Anser indicus/China/ZT-BTY1/1/2021(H5N1)	ZT-BTY1	PQRERRRKKR↓GLF	Red patches on the heart, trachea and stomach
A/Anser indicus/China/ZT-BTY2/2/2021(H5N1)	ZT-BTY2	PLREKRRKR↓GLF	No obvious lesion
A/Anser indicus/China/ZT-BTY3/3/2021(H5N1)	ZT-BTY3	PLREKRRKR↓GLF	No obvious lesion
A/Anser indicus/China/ZT-BTY4/4/2021(H5N1)	ZT-BTY4	PLREKRRKR↓GLF	Pancreatic lesions
A/Anser indicus/China/ZT-BTY5/5/2021(H5N1)	ZT-BTY5	PLREKRRKR↓GLF	Edema of the heart and liver, pancreatic lesions

Table S2. Homology of HPAI H5N1 eight genes from Anser indicus with the most similar sequences in the NCBI GenBank databases.

Yunnan H5N1 variants	Gene segment	Accession no.	Viruses in the NCBI database	Identity (%)
A/Anser indicus/China/ZT-BTY1/1/2021(H5N1)	PB2	OP597635.1	A/pelican/Tumen/932-1/2021(H5N1)	99.65
	PB1	OP597633.1	A/pelican/Tumen/1032-1/2021(H5N1)	99.56
	PA	OP597640.1	A/pelican/Tumen/932-1/2021(H5N1)	99.54
	HA	MW961428.1	A/chicken/Nigeria/VRD21-43/2021(H5N8)	99.16
	NP	OP030703.1	A/duck/Bangladesh/51601/2021(H5N1)	99.4
		MW961495.1	A/chicken/Nigeria/VRD21-98/2021(H5N1)	
	NA	OP597622.1	A/goose/Chelyabinsk/1341-3/2021(H5N1)	99.21
	M	OP597621.1	A/goose/Chelyabinsk/1341-3/2021(H5N1)	99.9
	NS	OP590537.1	A/pintail/Egypt/RA19853OP/2021(H5N1)	99.65
A/Anser indicus/China/ZT-BTY2/2/2021(H5N1)	PB2	OP597635.1	A/pelican/Tumen/932-1/2021(H5N1)	99.68
	PB1	OP597633.1	A/pelican/Tumen/1032-1/2021(H5N1)	98.56
	PA	OP597640.1	A/pelican/Tumen/932-1/2021(H5N1)	99.54
	HA	MW961428.1	A/chicken/Nigeria/VRD21-43/2021(H5N8)	99.18
	NP	OP030703.1	A/duck/Bangladesh/51601/2021(H5N1)	99.27
		MW961495.1	A/chicken/Nigeria/VRD21-98/2021(H5N1)	

	NA	OP590535.1	A/pintail/Egypt/RA19853OP/2021(H5N1)	98.79
	M	OP597621.1	A/goose/Chelyabinsk/1341-3/2021(H5N1)	99.9
	NS	OP590537.1	A/pintail/Egypt/RA19853OP/2021(H5N1)	99.53
A/Anser indicus/China/ZT-BTY3/3/2021(H5N1)	PB2	OP597635.1	A/pelican/Tumen/932-1/2021(H5N1)	99.55
	PB1	OP597633.1	A/pelican/Tumen/1032-1/2021(H5N1)	99.38
	PA	OP597640.1	A/pelican/Tumen/932-1/2021(H5N1)	99.54
	HA	MW961428.1	A/chicken/Nigeria/VRD21-43/2021(H5N8)	99.16
	NP	OP030703.1	A/duck/Bangladesh/51601/2021(H5N1)	99.4
		MW961495.1	A/chicken/Nigeria/VRD21-98/2021(H5N1)	
	NA	OP597622.1	A/goose/Chelyabinsk/1341-3/2021(H5N1)	99.12
	M	OP597621.1	A/goose/Chelyabinsk/1341-3/2021(H5N1)	99.69
	NS	OP590537.1	A/pintail/Egypt/RA19853OP/2021(H5N1)	99.53
A/Anser indicus/China/ZT-BTY4/4/2021(H5N1)	PB2	OP030699.1	A/duck/Bangladesh/51601/2021(H5N1)	99.51
	PB1	OP030700.1	A/duck/Bangladesh/51601/2021(H5N1)	99.5
	PA	OP597640.1	A/pelican/Tumen/932-1/2021(H5N1)	99.59
	HA	OP030702.1	A/duck/Bangladesh/51601/2021(H5N1)	99.39
	NP	OP030703.1	A/duck/Bangladesh/51601/2021(H5N1)	99.74
	NA	OP030704.1	A/duck/Bangladesh/51601/2021(H5N1)	99.7
	M	OP030705.1	A/duck/Bangladesh/51601/2021(H5N1)	99.69
	NS	OP030706.1	A/duck/Bangladesh/51601/2021(H5N1)	99.65
A/Anser indicus/China/ZT-BTY5/5/2021(H5N1)	PB2	OQ797589.1	A/northern pintail/duck/Egypt/2019 (H1N1)	98.5
	PB1	OQ793822.1	A/garganey/Egypt/MB-D-1323C/2017(H3N8)	98.57
	PA	MW188628.1	A/duck/Mongolia/447/2018(H4N6)	99.03
	HA	OP030702.1	A/duck/Bangladesh/51601/2021(H5N1)	99.76
	NP	MW935150.1	A/duck/Vietnam/HN5894/2019(H4N6)	98.94
	NA	OP030704.1	A/duck/Bangladesh/51601/2021(H5N1)	99.07
	M	OP030705.1	A/duck/Bangladesh/51601/2021(H5N1)	99.69
	NS	MN209303.1	A/duck/Bangladesh/36395/2018(H4N6)	99.53
